# Prognosis of patients with diffuse large B cell lymphoma not reaching complete response or relapsing after frontline chemotherapy or immunochemotherapy

**DOI:** 10.1007/s00277-014-2271-1

**Published:** 2014-12-14

**Authors:** Jordina Rovira, Alexandra Valera, Lluis Colomo, Xavier Setoain, Sonia Rodríguez, Alejandra Martínez-Trillos, Eva Giné, Ivan Dlouhy, Laura Magnano, Anna Gaya, Daniel Martínez, Antonio Martínez, Elías Campo, Armando López-Guillermo

**Affiliations:** 1 Hematology Department, Hospital Clínic, IDIBAPS, C/. Villarroel, 170, 08036 Barcelona, Spain; 2 Pathology Department, Hospital Clínic, IDIBAPS, University of Barcelona, Barcelona, Spain; 3 Nuclear Medicine Department, Hospital Clínic, Barcelona, Spain; 4 Radiology Department, Hospital Clínic, Barcelona, Spain

**Keywords:** Relapse/refractory, DLBCL, Immunochemotherapy

## Abstract

A retrospective study was performed to assess the outcome of patients with diffuse large B cell lymphoma (DLBCL) who did not achieve complete response or who relapsed before and after the use of rituximab. Clinical features and outcome of 816 (425 M/391 F; median age 63 years) patients diagnosed from 1991 to 2001 (pre-rituximab era, *N =* 348) and from 2002 to 2012 (rituximab era, *N =* 468) in a single institution were evaluated. Five hundred fifty-three patients achieved complete remission (CR), 57 partial response (PR), and 206 were refractory with a median overall survival of 15, 1.5, and 0.4 years, respectively. Patients receiving rituximab had lower risk of refractoriness or relapse. In primarily refractory and PR patients, there was not a difference in survival depending on whether patients received or not rituximab-containing frontline treatment. Early death rate was 11 %, including 3.6 % due to infectious complications. Rituximab did not modify these figures. In the relapse setting, 5-year survival from relapse was 25 % for patients who never received rituximab, 54 % for those who received rituximab only at relapse, and 48 % for those treated with immunochemotherapy both as frontline and at relapse. In conclusion, relapsed/refractory patients with DLBCL show poor prognosis despite the use of frontline immunochemotherapy. New therapeutic approaches are needed in this group of patients.

## Introduction

Diffuse large B cell lymphoma (DLBCL) is the commonest subtype of non-Hodgkin’s lymphoma accounting for approximately 30–50 % of cases [1]. DLBCL shows an aggressive behavior with a median survival of less than 1 year in untreated patients. Since the 1970s, the CHOP regimen (cyclophosphamide, adriamycin, vincristine, and prednisone) has been the standard treatment with 50 % of complete remissions (CR) and 30–40 % of long survivors [2]. Prognosis of DLBCL has considerably improved during the last decade, mainly due to the addition of rituximab, the first approved anti-CD20 monoclonal antibody, to chemotherapy (CT), known as immunochemotherapy. The addition of rituximab to firstline treatment improved CRs by 15–20 %, 5-year event-free survival (EFS) from 29 to 47 % in patients 60 to 80 years old and 3-year EFS or progression-free survival (PFS) from 59 to 79 % in patients aged 18 to 60 [3–9]. More importantly, immunochemotherapy significantly improved overall survival (OS) [3–9]. Therefore, rituximab-CT (R-CT) became the new standard of care by 2002. However, despite these notable advancements, there is still a considerable proportion of patients that are primarily refractory or experience short-term relapses impairing their possibilities of survival. For patients with chemosensitive disease, the standard treatment for relapsed/refractory DLBCL cases is salvage CT followed by autologous stem cell transplantation (ASCT) [10]. Patients who progress while receiving frontline therapy or those relapsing very early (the so-called primary chemorefractory patients) are less likely to respond to salvage treatment and therefore, have a clearly inferior PFS and OS than late relapses [11, 12]. After the PARMA study, several retrospective analyses have supported the role of ASCT in primarily chemorefractory cases [13, 14]. Typical salvage CT regimens are platinum based plus high-dose cytarabine, ifosfamide or gemcitabine. Currently, the use of immunochemotherapy at relapse is generalized, although the evidence to support this fact, particularly in R-CT refractory patients, is scarce. Moreover, it has been suggested that the prognosis of relapsed/refractory patients is worse nowadays than before rituximab [15, 16]. Whether the outcome of DLBCL patients not reaching CR or who eventually relapse has changed in the last decade with the use of frontline immunochemotherapy is still an open question.

In this setting, the aim of the present study was to assess the clinical features, response to salvage treatment, PFS and OS of patients with relapsed/refractory DLBCL before and after the addition of rituximab to firstline therapy in a single institution.

## Patients and methods

### Patients

Nine hundred eighty-three patients were consecutively diagnosed with DLBCL according to the WHO classification [17], in a single institution between 1991 and 2012. The only criterion to include patients was the availability of histological material. Patients with post-transplant lymphoproliferative disorders (*N* = 9), immunodeficiency-associated (*N* = 95), central nervous system (*N* = 39), primary effusion (*N* = 1), and primarily mediastinal lymphomas (*N* = 37) were excluded. Thus, 816 patients were the subject of the present study. Patients were divided in two groups: 348 patients (42.6 %) diagnosed during the pre-rituximab era (1991–2001) (pre-R) and 468 patients (57.4 %) during the rituximab period (2002–2012) (R). The main characteristics of patients are described in Table 1. The median age of patients was 63 years (range 14–94) and the male/female distribution 425 (52 %)/391 (48 %). Most variables showed no significant differences through time, but during the last decade, the median age was higher and the proportion of extranodal involvement lower. The first cohort of patients received chemotherapy (CT) and the second one immunochemotherapy (R-CT) unless intolerance or toxicity (*N = 3)*. The frontline regimens slightly varied over time and could be summarized as curative intention (CHOP or CHOP-like) or palliative intention (COP) depending on the use or not of adriamycin. The proportion of patients treated with curative intention was higher in the second cohort than that in the first one (85 vs 78 %, respectively; *P* = 0.01).

**Table 1:** Clinical characteristics at diagnosis of 816 patients with diffuse large B cell lymphoma

	All	1991–2001	2002–2012
	*N* (%)	*N* (%)	*N* (%)
Patients	816 (100)	348 (43)	468 (57)
Gender			
Male	425 (52)	167 (48)	258 (55)*
Female	391 (48)	181 (52)	210 (45)
Age			
≤60	364 (45)	174 (50)	190 (41)*
>60	452 (55)	174 (50)	278 (59)
B symptoms	319 (40)	132 (38)	187 (41)
ECOG performance status			
<2	465 (59)	191 (55)	274 (62)
≥2	319 (41)	153 (44)	166 (38)
Bulky disease (>7 cm)	226 (29)	102 (30)	124 (29)
Primarily extranodal	290 (35)	140 (40)	150 (32)*
Extranodal involvement	525 (64)	242 (70)	283 (60)*
BM involvement	126 (16)	65 (19)	61 (13)*
Ann Arbor stage			
I–II	380 (47)	175 (51)	205 (45)
III–IV	426 (53)	171 (49)	255 (55)
High serum LDH	395 (52)	181 (55)	214 (50)
High serum β2m	280 (46)	103 (45)	177 (47)
IPI risk group			
Low	271 (35)	120 (36)	151 (34)
Low intermediate/high intermediate	326 (42)	127 (38)	199 (45)
High	179 (23)	83 (25)	96 (21)

*ECOG* Eastern Cooperative Oncology Group, *LDH* lactate dehydrogenase, *β*_*2*_ *m* β_2_-microglobulin, *BM* bone marrow

*P < 0.05

The study was performed according to the guidelines of the Ethic Committee of the Hospital Clínic of Barcelona. Informed consent to use the clinical data was obtained in accordance with the Declaration of Helsinki.

### Staging and treatment

The following variables were recorded and analyzed: (i) demographic data, performance status according to the Eastern Cooperative Oncology Group (ECOG) scale, presence of B symptoms (fever, night sweats, weight loss), and bulky disease (defined as a tumor diameter >7 cm); (ii) hematological and biochemical parameters: blood cell counts, hemoglobin, serum lactate dehydrogenase (LDH), and β_2_-microglobulin (β_2_m) levels; (iii) tumor extension data: nodal and extranodal involvement, number of extranodal involved sites, palpable splenomegaly or hepatomegaly, bone marrow infiltration and Ann Arbor Stage; (iv) the International Prognostic Index (IPI) [18]; (v) treatment; response to treatment, relapse, cause of death; (vi) appearance of secondary neoplasias.

### Response to therapy and outcome

The criteria to assess response were those of Cheson 1999, based on CT scan. Thus, CR was defined as the total disappearance of tumor masses and disease-related symptoms as well as the normalization of the initial abnormal test for at least 1 month. CR/unconfirmed (CRu) included a residual lymph node mass greater than 1.5 cm that regressed more than 75 % and individual nodes that were previously confluent that regressed more than 75 %. Partial response (PR) was considered when tumor mass or organ infiltration decreased by at least 50 % along with the disappearance of disease-related symptoms. CR and CRu patients were analyzed together. In addition, since 2005 when PET scan became available in our institution, this procedure was used to assess response, and therefore, patients with residual masses were considered in PR when PET scan was positive and in CR when PET scan was negative [19]. Patients not included in these categories and early deaths were considered nonresponders. Early death was defined as those patients dying within the 4 months after diagnosis. Disease relapse or progression was defined as the appearance of new symptoms or signs of the disease as demonstrated by lymph node biopsy or other appropriate studies. OS and PFS were calculated according to standard definitions [19].

### Statistical methods

Differences among the subgroups of patients were assessed by using the Chi-square test (two-tailed), the Student’s *t* test, or nonparametric tests when necessary. The actuarial survival analysis was performed by the Kaplan and Meier method and differences assessed by the log-rank test. To evaluate the prognostic impact of different variables in response to salvage therapy, PFS, and OS, multivariate analyses were performed with the stepwise proportional hazards model (Cox model) [20]. *P* values <0.05 were considered statistically significant.

## Results

### Response to frontline treatment and outcome

After therapy, 553 patients (68 %) achieved CR, 57 PR (7 %), and 206 did not respond (25 %). As expected, the CR rate was higher in patients treated with R-CT compared to those receiving CT alone (71 vs 64 %, respectively; *P* = 0.036) and particularly in patients treated with curative intention (77 vs 68 %, respectively; *P* = 0.009). In addition, the proportion of primary refractory cases was significantly lower in the R-CT subgroup (29 vs 23 %, respectively; *P* = 0.05), specifically in patients treated with curative intention (24 vs 15 %, respectively; *P* = 0.009). Those patients (*N =* 263) who did not achieve CR were older and more often had poor ECOG performance status, B symptoms, advanced Ann Arbor stage, bulky disease, extranodal involvement, bone marrow and CNS infiltration, high serum LDH and β_2_m, high-risk IPI and received palliative approach as compared with those achieving CR (*P* ≤ 0.008 in all cases).

**Fig. 1:**
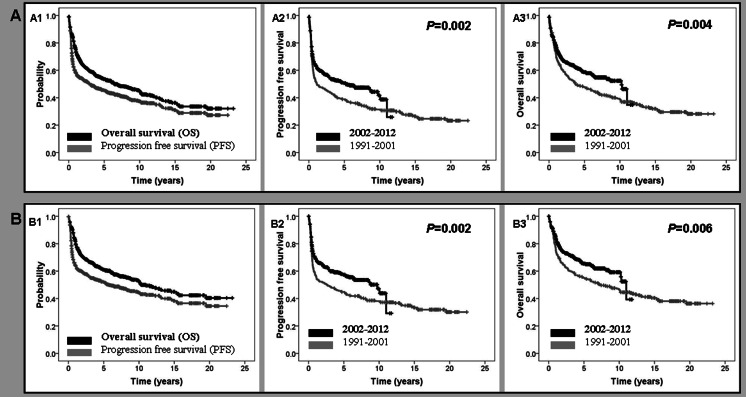
Outcome of the whole series of patients with diffuse large B cell lymphoma (**a**) and of those treated with curative intention (**b**). Overall survival (OS) and progression-free survival (PFS) of the subgroups (**a1**, **b1**). PFS according to the year of diagnosis (before and after December 2001) (**a2**, **b2**). OS according to the year of diagnosis (before and after December 2001) (**a3**, **b3**)

Median follow-up for surviving patients was 6.5 years (range 0.02–23.2). One hundred fifty out of 553 patients in CR eventually relapsed. Five-year PFS was 45.5 % (95 % CI 44.4–46.6 %), with significant differences between the pre-R and R period (5-year PFS 39 vs 51 %, respectively; *P* = 0.002) (Table 2). This difference was also observed in patients treated with curative intention (5-year PFS 45 vs 57 %, respectively; *P* = 0.002). Four hundred and nineteen patients died during the follow-up with a 5-year OS of 54 % (95 % CI 50.2–57.2 %), with a significant difference between pre-R and R eras (5-year OS 48 vs 59 %, respectively; *P* = 0.004). Once again, the difference was maintained in patients treated with curative intention (5-year OS 55 vs 65 %, respectively; *P* = 0.006). A multivariate analysis showed that in a model of 582 patients, IPI (*P* < 0.0001, HR 1.9, 95 % CI 1.7–2.3), use of rituximab (*P* < 0.0001, HR 0.5, 95 % CI 0.4–0.6) and bulky disease (*P* = 0.048, HR 1.3, 95 % CI 1.0–1.6) were the most important variables affecting OS. PFS and OS curves of the whole series and of those patients treated with curative intention are shown in Fig. 1. The cause of death was lymphoma in 83 % of cases which was similar in both groups. Twenty-seven second neoplasias (3.4 %) were detected during the follow-up with no significant differences between the two subgroups.

**Table 2:** Outcome of 816 patients with diffuse large B cell lymphoma

	Whole series	1991–2001	2002–2012	*P* value
	*N* = 816	*N* = 348	*N* = 468	
Treatment response, *N* (%)				0.036
CR	553 (68)	222 (64)	331 (71)	
PR	57 (7)	26 (7)	31 (7)	
No response	206 (25)	100 (29)	106 (23)	
PFS at 5 years (%)	45.5	39	51	0.002
OS at 5 years (%)	53.7	48	59	0.004
Relapse after CR, *N* (%)	150 (27)	81 (36)	69 (21)	<0.0001
Survival from relapse at 5 years (%)	29	29	35	NS
Relapse ≤2 years from CR (%)	20	21	19	NS
Relapse >2 years from CR (%)	46	44	48	NS

*CR* complete response, *PR* partial response, *PFS* progression-free survival, *OS* overall survival

### Salvage treatment in primary chemorefractory patients

**Fig. 2:**
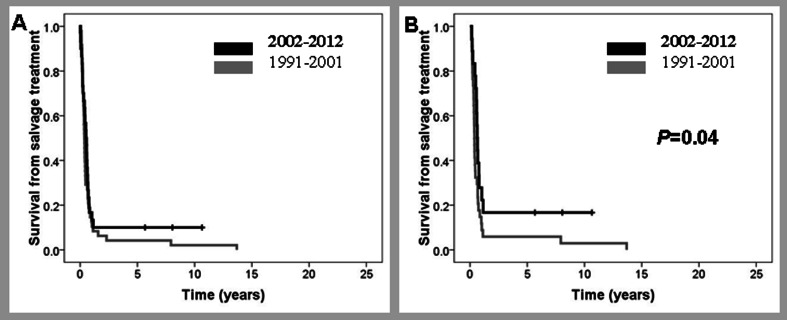
Survival from salvage treatment of frontline chemorefractory patients diagnosed before or after December 2001. **a** All patients; **b** only patients with curative intention to salvage treatment

Ninety-two out of 206 patients (45 %) who did not reach a response died within 4 months from diagnosis, including 10 patients who were never treated. These early death rates in patients receiving CT or R-CT were 35/348 (10 %) versus 57/468 (12 %), respectively. Infectious complications were the ultimate cause of death in 30 cases (3.2 vs 4 % in pre-R and R era, respectively), irrespective of the possible response of the disease. One hundred fourteen patients surviving more than 4 months were primary refractory to treatment; the median OS of this group was 0.75 years (Fig. 2a). Sixty-one of these patients (31 pre-R; 30 R) received only palliative measures mainly due to age and/or poor ECOG performance status, and all of them died between 4 and 44 months from diagnosis. Salvage treatment was administered to 53 patients (34 and 19 in the pre-R and R era, respectively). In the pre-R era, only one patient achieved CR (3 %) and three PR (9 %) whereas in the R era, three patients achieved CR (16 %) and five PR (26 %) (*P* = 0.027). One patient in PR in the pre-R era received allogeneic stem cell transplantation (Allo-SCT). This patient died in CR at 7.2 years after transplant due to esophagus carcinoma. In the R era, three patients received an ASCT and one an Allo-SCT. The only patient in the pre-R era who achieved CR died due to a stroke 14 years from the assessment of response. Three patients in the R era were long survivors. Overall, the median survival after salvage therapy of those patients treated with curative intention was 0.46 years (0.39 years in pre-R era vs 0.64 years in R era; *P* = 0.044) (Fig. 2b). Responses and survival after salvage treatment are summarized in Table 3.

**Table 3:** Outcome of 668 patients with diffuse large B cell lymphoma with chemorefractory disease, partial remission (PR), or relapse, treated with curative intention

	Primary chemorefractory		PR		Relapsed	
	1991–2001 *N = 34*	2002–2012 *N = 19*	1991–2001 *N* = 18	2002–2012 N = 22	1991–2001 *N* = 57	2002–2012 N = 34
Response to salvage treatment, *N* (%)	1 (3)	3 (17)	5 (28)	11 (50)	29 (51)	20 (59)
CR	3 (9)	5 (26)	4 (22)	3 (14)	6 (10)	5 (15)
PR No response	30 (88)	11 (58)	9 (50)	8 (36)	22 (39)	9 (26)
ASCT (%)	3 (9)	4 (21)	4 (22)	9 (41)	21 (37)	9 (26)
5-year survival from progression (%)	6	15*	26	38	33	42

*CR* complete response, *PR* partial response, *ASCT* autologous stem cell transplantation

*P < 0.05

### Salvage treatment in patients in PR

**Fig. 3:**
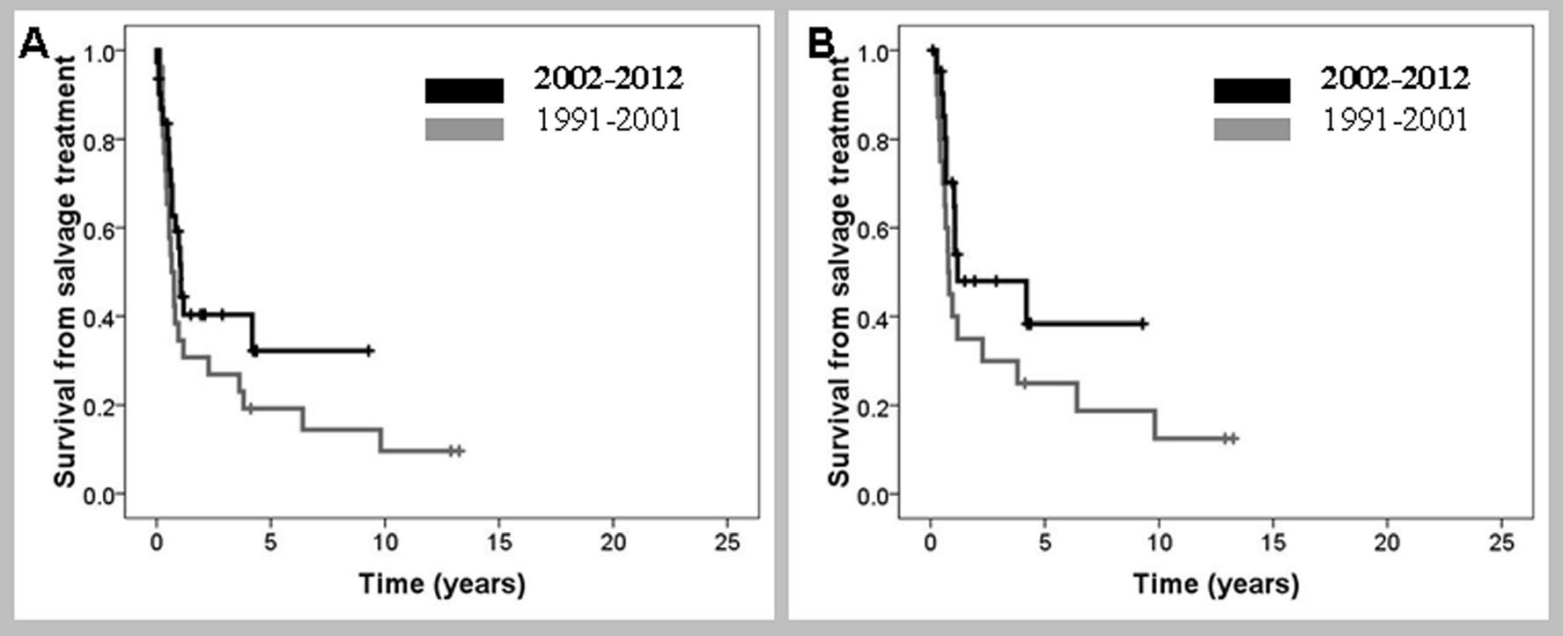
Survival from salvage treatment of patients in partial remission after frontline therapy diagnosed before or after December 2001. **a** All patients; **b** only patients with curative intention to salvage treatment

Fifty-seven patients were in PR after induction therapy, 26/348 (7.5 %) treated with CT, and 31/468 (7 %) with R-CT. When restricted to curative treatment, the figures were 22/271 (8.1 %) and 29/397 (7.3 %). Fifteen of the 57 patients (6 pre-R; 9 R) received only palliative measures, whereas 42 (20 pre-R; 22 R) received ESHAP salvage therapy (etoposide, methylprednisolone, high-dose cytarabine, and cisplatin). Only two patients in the pre-R era received rituximab as part of salvage therapy, whereas all the cases in the R era were treated with R-ESHAP. CR rates were 25 % (5 out of 20) and 50 % (11 out of 22) in the pre-R and R era, respectively (*P* = 0.09). ASCT was performed in four pre-R and nine R patients. Long survival was 11.5 % (*N =* 3) and 35 % (*N =* 11), respectively (*P* = 0.03). Overall, the median survival after salvage treatment was 1 year, with a 5-year survival from rescue therapy of 19 vs 32 % for patients in the pre-R and R eras (Fig. 3a). Regarding patients treated with curative intention, the 5-year survival after salvage treatment was 26 vs 38 % (Fig. 3b and Table 3). Among four patients who underwent ASCT in the pre-R era, two achieved CR and two died early after ASCT due to cardiogenic shock and infection. One patient in CR died shortly afterward of disease progression. In the R era, six out of nine patients achieved CR, three died of disease progression, and two relapsed and were treated with a reduced intensity Allo-SCT. One of them died due to infectious complication, and the other patient is alive after 10 years.

### Salvage treatment in relapsed patients

One hundred and fifty out of 553 patients (27.1 %) in CR eventually relapsed (Table 2). Such proportion was 36 and 21 % during the pre-R and R era, respectively (*P* < 0.0001). In the 70 patients in whom a new biopsy was performed, the histology at relapse corresponded to DLBCL in 95 % of cases and to a downgrade relapse in 5 % (all follicular lymphoma). Relapse was primarily extranodal in 58 cases. No differences in the localization relapse or histology were observed between the pre-R and R eras. During the last decade, the CR duration (time from CR achievement to relapse) has been significantly longer than that in the pre-R era (median time 0.75 vs 1.5 years; *P* = 0.027). Finally, more relapses occurred in older patients (>70 years), namely, 17 % pre-R vs 39 % R era (*P* = 0.003).

Salvage treatment consisted of CT or R-CT followed by ASCT for those patients <65–70 years of age, good performance status and achieving PR or CR after rescue. This intensive approach was performed in 82 % of pre-R patients and 56 % of R patients. This difference was because the second group was older. On the other hand, 11 of 76 patients initially treated only with CT received rituximab at relapse, whereas all patients treated in the R era received rituximab at relapse.

**Fig. 4:**
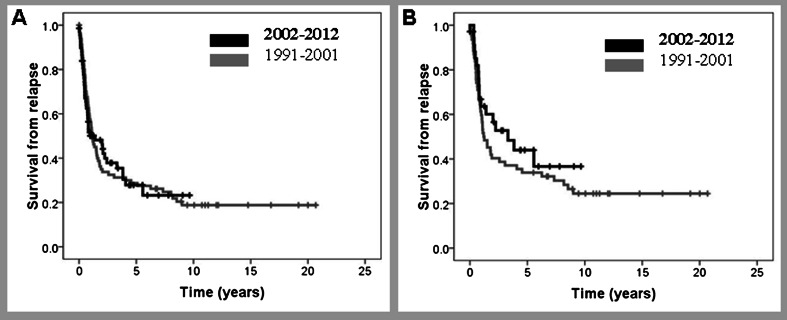
Survival from relapse diagnosed before or after December 2001. **a** All patients; **b** only patients with curative intention to salvage treatment

**Fig. 5:**
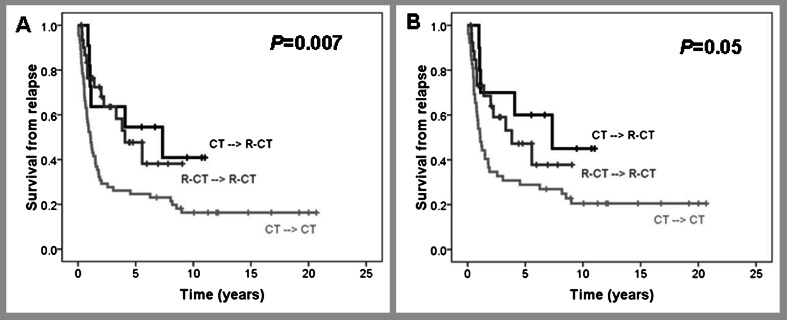
Survival from relapse according to rituximab treatment at diagnosis or relapse. *CT → R-CT* chemotherapy at diagnosis and immunochemotherapy at relapse; *R-CT → R-CT* immunochemotherapy both at diagnosis and at relapse; *CT → CT* chemotherapy both at diagnosis and at relapse; **a** all cohort; **b** only patients treated with curative intention at relapse

CR rates after salvage therapy were 42 versus 39 % in the pre-R and R era, respectively. In those patients treated with curative intention, CR rates were 51 versus 59 % (Table 3). Median survival from relapse (SFR) was 1.12 years, with a 29 % pre-R versus 28 % R 5-year SFR in pre-R and R eras (Fig. 4a). Median SFR of patients treated with curative intention was 2 years, whereas 5-year SFR was 33 versus 42 % in the pre-R and R eras, respectively (Fig. 4b). Variables at relapse predicting shorter SFR were older age (5-year SFR of 39 versus 22 % for patients <60 and ≥60 years, respectively, *P* = 0.04) and CNS relapse (5-year SFR of 37 and 18 % patients with or without CNS involvement, respectively, *P* = 0.02). A trend to shorter SFR was observed in those patients relapsing during the first 2 years after CR achievement (5-year SFR of 20 versus 46 % for patients relapsing ≤2 years or later, *P* = 0.07). SFR is shown in Fig. 5 according to the use of rituximab in front line and at relapse. Five-year SFR was 25, 54, and 48 % for patients who never received rituximab, those who received the drug only at relapse and those treated with rituximab both in front line and rescue regimen, respectively (*P* = 0.007). Such differences were maintained in patients treated with curative intention (Fig. 5b). A multivariate analysis showed that in a model of 69 patients, age at relapse >70 years (*P* = 0.023, HR 2.1, 95 % CI 1.1–3.9) and CNS involvement (*P* = 0.04, HR 2.1, 95 % CI 1.0–4.4) were the most important variables to predict SFR.

## Discussion

Since the 1970s, the treatment of patients with DLBCL has been based on CT. The addition of rituximab to CT dramatically improved the outcome of these patients, as demonstrated in clinical trials and in retrospective population-based studies [3–9]. Thus, immunochemotherapy is currently the gold standard treatment for any CD20-positive DLBCL [3]. Despite this advance, a considerable number of patients will experience early failure, partial response, or relapse after initial rituximab-CT (R-CT). Nowadays, the outcome of relapsed/refractory (R/R) patients is still poor. Some evidence suggests that patients treated with R-CT could be more resistant to salvage therapy than before the use of rituximab. In this setting and in order to highlight the challenges faced between the pre-rituximab era and the current immunochemotherapy, the aim of our study was to evaluate the characteristics and outcome of those patients with R/R DLBCL after frontline treatment in a single institution. Published data concerning salvage treatment is most often based on highly selected series of patients in whom intensive treatment is possible [4, 6, 10–12, 21–25]. Such cases are not representative of the entire population of patients who fail to initial therapy. An analysis of an unselected series of nonresponders, as herein done, can offer a more realistic view of the efficacy of salvage treatment and the real outcome in the general population.

Patients dying during induction treatment constitute a particular category of nonresponders. In the present study, 92 patients (11 % of the overall series and 45 % of nonresponders) died during the induction period. It is often difficult to distinguish between toxic death and disease progression as causes of death. In our series, 3.7 % of patients died due to infectious complications. No differences were observed between patients receiving CT or R-CT. This mortality rate is similar to that reported in the literature [3, 5, 26]. After excluding early deaths, primary refractory patients were considered as a different category which accounted for 14 % of the present series. More than half of them received only palliative measures mainly due to older age and/or poor performance status. All of them died within the next 3 years. Only 9 % of rescued patients achieved CR, although this proportion has improved in the last decade. These data apparently contrast with those reported in the pre-R era with overall response rates between 39 and 69 % including CR rates of 18–48 % [27–30]. However, in those series, the proportion of primary refractory patients was very low. In fact, our results in the R era are similar to those reported in recent studies with an overall response of 23–33 % and a CR of 6–8 % [11, 31]. These differences could be related to the definition of primary refractory disease and the selection of patients. Thus, some series included patients in PR and refractory as a whole. Our definition of refractory disease was strict and included only patients with stable or progressive disease. In summary, in our study, less than 10 % of the refractory patients responded to salvage treatment and were candidate to an eventual intensification. Therefore, it is clear that all refractory patients should be considered for clinical trials with new drugs and novel mechanisms of action.

In contrast to other studies, patients in PR after frontline treatment were grouped separately. The definition of PR can be a problematic one. Nowadays, thanks to PET scan, it is easier to define PR and to distinguish this situation from CRu [19]. In our series, the number of patients in PR could have been overestimated before the PET scan availability. Since then, patients with residual masses were considered in PR when PET scan was positive and CR when PET scan was negative. Salvage treatment followed by ASCT is the standard of care for patients in PR. However, this approach is based in old retrospective studies, and there is some controversy since prospective trial data supporting the use of ASCT is lacking, particularly in the R-CT era [13, 14, 23, 32]. In the current study, PR patients represented 7 % of all series and showed a considerably better response to salvage treatment and outcome than primary refractory patients. When comparing data between the pre-R and R era, a slight improvement in CR rate and outcome after rescue did not reach statistical significance probably due to the low number of patients. In addition, a higher proportion of patients in this category may benefit from ASCT [15, 16, 25, 31, 33–36]. In this regard, it is of note that 15 of the 75 patients (20 %) could receive only palliative measures due to their older age or poor performance status. Conversely, despite the relatively high CR rate, long-term disease-free survival is rather modest with less than one third of patients being cured [25, 26, 31].

The last category is that of patients relapsing after having achieved CR. In recent studies, data indicates that the addition of rituximab to CT implies a substantial reduction of relapse with rates ranging from 20 to 35 % [8, 21, 24]. We have corroborated this finding with relapsing rates of 36 versus 21 % for patients receiving CT or R-CT, respectively. After the PARMA trial, the standard approach for relapsed patients (if eligible) is salvage therapy followed by ASCT. Fifty-three percent of OS is expected in sensitive patients [10]. The only prospective randomized trial of patients with DLBCL in first relapse or primary refractory in the rituximab era is the CORAL, in which 396 patients were randomized in 2 groups of salvage treatment: R-ICE (rituximab, ifosfamide, carboplatin, etoposide) versus R-DHAP (rituximab, dexamethasone, high-dose citarabine, cisplatinum) followed by ASCT in responders. No difference in outcome between the groups was recorded, and a 10 % improvement in terms of outcome was observed with respect to the pre-R era [15, 34–36]. However, Gisselbrecht et al. published that chemotherapy sensitivity before ASCT positively affected response rates, whereas the use of rituximab in first-line treatment had negative effect in those patients relapsing within 12 months after initial CR [15]. The role of rituximab in the second-line setting in refractory patients or in patients whose lymphoma progressed on R-CHOP is also unclear. Current information about the utility of rituximab as part of salvage treatment comes from retrospective analyses [6]. Unfortunately, it does not seem likely that prospective studies are planned to assess this issue. The addition of rituximab to second-line CT followed by ASCT has significantly improved PFS in patients not previously exposed to rituximab [25]. We have also observed that rituximab-naïve patients responded better to salvage treatment followed by ASCT compared to those patients treated both times with rituximab-containing regimens and to those who never received rituximab. This latter group had the worst prognosis. CR duration has been considered one of the most important prognostic factors for outcome in relapsed patients [37]. Interestingly, in our series, patients relapsing after R-CT showed a longer CR duration than those relapsing after CT (1.5 vs 0.7 years). This could be in part the reason why the short duration of CR had a modest impact on survival in the present series.

Based on the PARMA trial, ASCT is the standard of care in R/R patients with DLBCL responding to salvage treatment. However, no other randomized information is available, particularly in the R era. We must be cautious with results from clinical trials as from other published data concerning salvage treatment approaches since there are most often based on highly selected series of patients in whom intensive treatment is possible [4, 6, 10–12, 21–25]. In fact, the median age of those series is low. Most likely such patients who fail initial therapy are not representative of the entire population. In the present series which includes the whole population of R/R patients, it is of note that only a minority of those could benefit from intensified treatments. Therefore, the proportion of cases transplanted was 22, 23, and 9 % for relapsed, PR, and refractory patients, respectively. Moreover, the use of rituximab does not seem to have changed this situation.

Prognosis of DLBCL has considerably improved during the last 10 years. However, an important proportion of patients are not yet cured, and the treatment of relapses remains unsatisfactory as shown in the present series. Achieving a sustained CR after first-line therapy is an essential goal. A better biological understanding of early relapses and primarily refractory lymphomas is needed, as well as the development of new drugs with different mechanisms of action for R/R patients.
